# Revised Vertebral Count in the “Longest-Necked Vertebrate” *Elasmosaurus platyurus* Cope 1868, and Clarification of the Cervical-Dorsal Transition in Plesiosauria

**DOI:** 10.1371/journal.pone.0070877

**Published:** 2013-08-05

**Authors:** Sven Sachs, Benjamin P. Kear, Michael J. Everhart

**Affiliations:** 1 Engelskirchen, Germany; 2 Department of Earth Sciences, Uppsala University, Uppsala, Sweden; 3 Sternberg Museum of Natural History, Fort Hays State University, Hays, Kansas, United States of America; University of Pennsylvania, United States of America

## Abstract

Elasmosaurid plesiosaurians are renowned for their immensely long necks, and indeed, possessed the highest number of cervical vertebrae for any known vertebrate. Historically, the largest count has been attributed to the iconic *Elasmosaurus platyurus* from the Late Cretaceous of Kansas, but estimates for the total neck series in this taxon have varied between published reports. Accurately determining the number of vertebral centra vis-à-vis the maximum length of the neck in plesiosaurians has significant implications for phylogenetic character designations, as well as the inconsistent terminology applied to some osteological structures. With these issues in mind, we reassessed the holotype of *E. platyurus* as a model for standardizing the debated cervical-dorsal transition in plesiosaurians, and during this procedure, documented a “lost” cervical centrum. Our revision also advocates retention of the term “pectorals” to describe the usually three or more distinctive vertebrae close to the cranial margin of the forelimb girdle that bear a functional rib facet transected by the neurocentral suture, and thus conjointly formed by both the parapophysis on the centrum body and diapophysis from the neural arch (irrespective of rib length). This morphology is unambiguously distinguishable from standard cervicals, in which the functional rib facet is borne exclusively on the centrum, and dorsals in which the rib articulation is situated above the neurocentral suture and functionally borne only by the transverse process of the neural arch. Given these easily distinguishable definitions, the maximum number of neck vertebrae preserved in *E. platyurus* is 72; this is only three vertebrae shorter than the recently described *Albertonectes*, which together with *E. platyurus* constitute the “longest necked” animals ever to have lived.

## Introduction

The Late Cretaceous plesiosaurian (Plesiosauria, Sauropterygia) *Elasmosaurus platyurus* from the Lower Campanian Sharon Springs Formation of western Kansas represents one of the most commonly reconstructed Mesozoic fossils. Its global fame has stemmed from the classical plesiosaurian neck, which is exceptionally long in *E. platyurus* and actually manifests amongst the highest number of individual cervical vertebrae found in any living or extinct vertebrate [1]. Nevertheless, the holotype specimen (Academy of Natural Sciences of Philadelphia [ANSP] 10081) of *E. platyurus* is also widely recognized as a subject of controversy, which began with its inaugural presentation at the March 24^th^ 1868 meeting of the ANSP by the eminent palaeontologist Edward Drinker Cope ([2]: p. 92). Cope initially characterized *E. platyurus* by the “enormous length of [it’s] tail, and the relatively shorter cervical region” [2]. He also specifically documented a total of 72 (68½ preserved +3½ missing) “caudal” vertebrae in his self-published “pre-print” released in August of the following year ([3]: p. 49). Cope further suggested that the total vertebral count of *E. platyurus* was 121, including a supplementary complement of 17½ vertebrae that he thought had been lost [3]. However, nearly 12 months later at the March 8^th^ 1870 meeting of the ANSP, Joseph Leidy ([4]: p. 9) pointed out that Cope had “described the skeleton [of *E. platyurus*] in a reversed position to the true one”, and formally published a report to that effect in the *American Journal of Science* [5]; the note stated that there were 72 vertebrae in the cervical series, ending where the first transverse process shifted location onto the neural arch. Contrary to popular accounts [6], [7], the infamous claim that Othniel Charles Marsh was the first to identify Cope’s blunder is incorrect [8]. Marsh was present at the March 8^th^ 1870 ANSP meeting (see [9]), but it was almost 20 years before he was anecdotally quoted in a newspaper article [10] as having discovered Cope’s error. In fact, Cope had already published a reply to Leidy in 1870 [11], and petulantly pointed out some of Leidy’s own mistakes when describing plesiosaurian vertebrae. Marsh, on the other hand, never wrote a single paper on the plesiosaurian fossils from Kansas, even though a number of specimens were available to him in the Yale Peabody Museum, including several that he had collected himself [12], [13], [14].

Cope attempted to recall the distributed copies of his original 1868 article, and hurriedly re-named the “caudals” of *E. platyurus* as cervicals ([15]: p. 49). He nonetheless held to his conviction that the tail was “a powerful swimming organ” ([15]: p. 54), and envisaged an additional 26 caudal vertebrae that he considered were missing. This amendment increased the total estimate to 147 (103½ preserved +43½ missing), but the “½” vertebra repeatedly listed by Cope [3], [15] inexplicably disappeared from later accounts. Indeed, the maximum cervical number attributed to *E. platyurus* has varied substantially in subsequent studies. For example, Williston ([16]: p. 225) reported 76 cervicals and three pectorals, the latter term defining the transitional morphotype interpolated between the cervicals and dorsals, and reckoned that the neck of *E. platyurus* was 23 ft (7 m) long. Welles ([17]: p. 185) alternatively listed, without explanation, 134 vertebrae including 74 cervicals and 3 pectorals, but reiterated Williston’s [16] proposed maximum body length of “42 ft” (∼13 m). Welles ([18]: p. 22) later revised his cervical count to 74 based on Cope’s drawing of the skeleton ([15]: pl. 2); despite this figure being incongruous with the accompanying table ([15]: p. 49). Welles ([19]: p. 53) later modified his conclusion following examination of the original remains, identifying “71 cervicals, 5 pectorals, 5 (+13 missing) dorsals, 6 sacrals and 16 (+5 missing) caudals” (total = 121), and noted “two [contrasting] sets of numbers [affixed to the holotype], one printed and the other inked” ([19]: p. 54; see also [6]: p. 148). We suspect that these labels were created by Cope (printed paper tabs) and Williston (inked) to illustrate their preferred sequential ordering of the vertebral column; however, Welles [19] is also known to have re-itemized the specimen himself [6].

Other appraisals of the *E. platyurus* holotype ANSP 10081 have further refined its vertebral groupings: Storrs [13] recorded 71 cervicals, five pectorals, five dorsals, six sacrals, and 16 caudals; Sachs [1] differed in recognizing three pectorals, seven dorsals ([1]: Table 2), four sacrals, and 18 caudals (note here that in fig. 5A of [1] the first pectoral has been called the last cervical, and the first dorsal was not described by [1]). Carpenter [12] increased the cervical count to 72 by redefining the nomenclature for plesiosaurian vertebrae to exclude the long-standing term “pectoral” (although the disposition of the remaining pectoral vertebrae of ANSP 10081 was not specified). Until very recently [20], this “final” count of 71 cervical vertebrae (or 72 *sensu* [12]) has remained the highest number reported from any plesiosaurian skeleton.

Everhart [21] described additional elements of a single elasmosaur specimen compatible with ANSP 10081 in collections of the Cincinnati Museum Center ([CMC] VP6865), Sternberg Museum of Natural History (Fort Hays State University [FHSM] VP-398), and University of Kansas ([KUVP] 129744), but there was another partial cervical centrum in the ANSP that was not stored with the vertebrae of the holotype – this might correspond to the missing “½” vertebra of Cope [3], [15]. If correct, this alters the total number of preserved vertebrae to 104 [21], and impacts on reinterpretations of neck length in this iconic taxon. Moreover, the osteological terminology applied to the transitional pectoral series in plesiosaurians has become increasingly convoluted, with arbitrary renaming of various components as cervicals [12], dorsals [22], or cervicals and dorsals [20]. Furthermore, incorrect directional nomenclature has fallen into common usage, and we therefore apply anglicized versions of “cranialis” and “caudalis” instead of “anterior” and “posterior” when referring to vertebral structures, as recommended by the *Nomina Anatomica Veterinaria* 2012 (www.wava-amav.org). In light of these compounding inconsistencies, we returned to the key specimen from which this confusion commenced, ANSP 10081, with the aim of standardizing the phylogenetically pertinent terminological differentiation of pectoral vertebra morphology in Plesiosauria.

## Attribution of Cope’s “Re-discovered” Cervical Centrum

**Figure 1:**
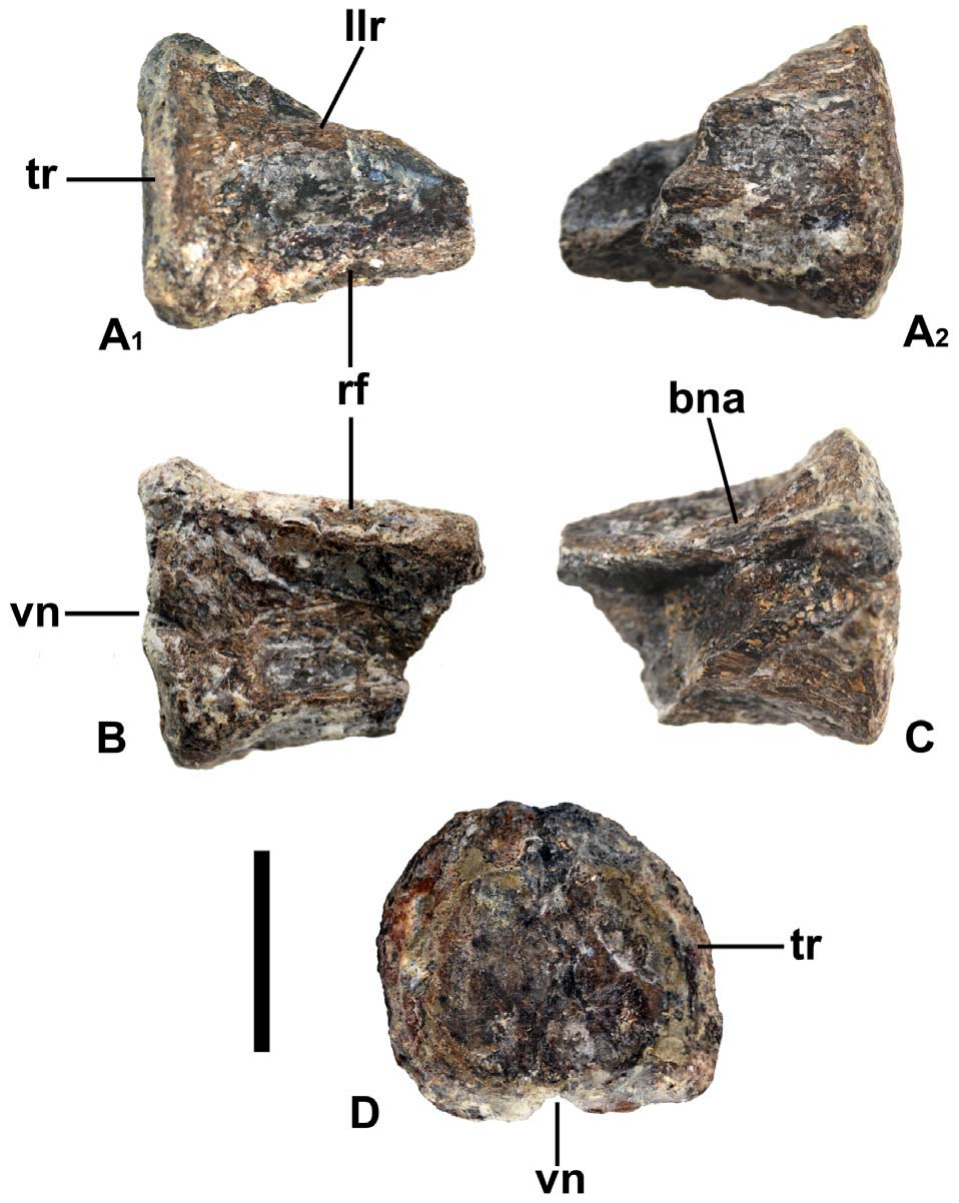
Cervical vertebra fragment from the ANSP collection. A1. and A2. lateral view, B. ventral view, C. dorsal view, D. articular view. **Abbreviations. bna** = base of the neural arch, **llr** = lateral longitudinal ridge, **rf** = single rib facet, **tr** = thickened rim, **vn** = ventral notch. Scale bar equals 3 cm.

**Figure 2:**
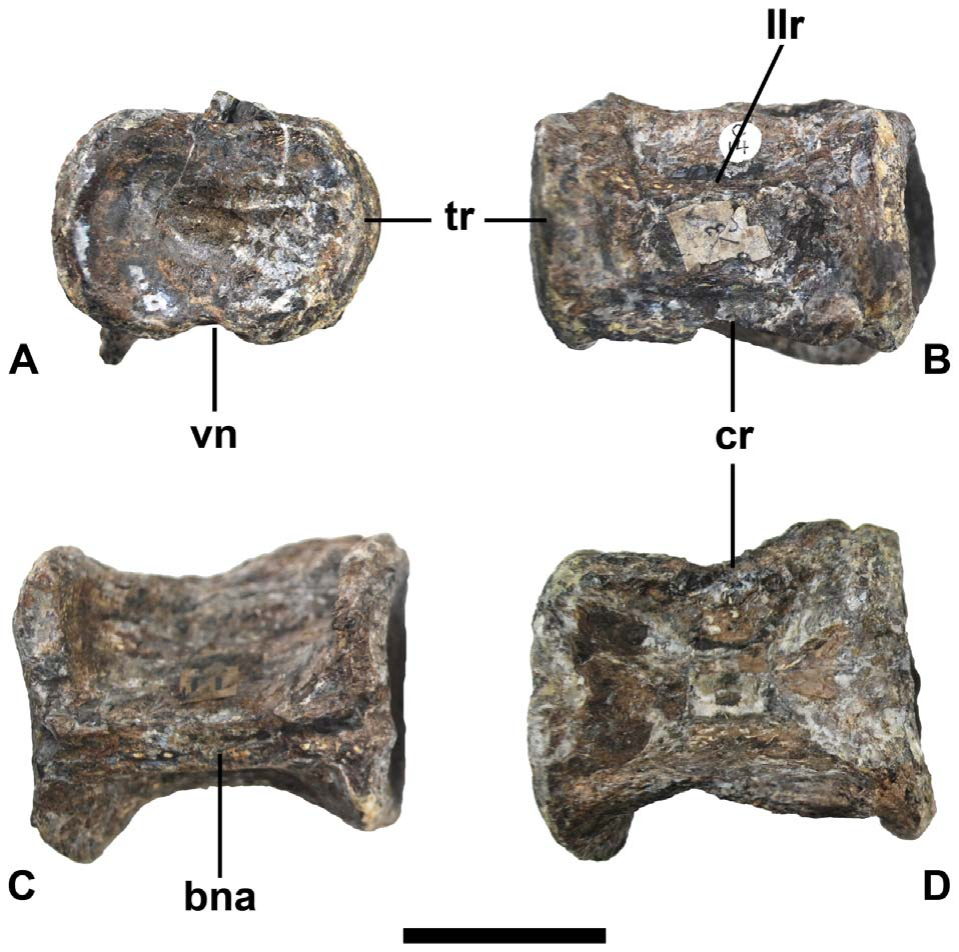
Craniad cervical vertebra of *Elasmosaurus platyurus* (ANSP 10081). A. articular, B. lateral, C. dorsal and D. ventral view. **Abbreviations.** **bna** = base of the neural arch, **cr** = cervical rib, **llr** = lateral longitudinal ridge, **tr** = thickened rim, **vn** = ventral notch. Scale bar equals 3 cm.

Most of the vertebral remains (103 isolated or articulated centra with remnants of the neural arches) cataloged under ANSP 10081 were displayed together for many years [6], but are presently housed with other referred fragments on a common shelf within a single storage cabinet. Each vertebra is marked with numbers assigned by previous researchers (see [21]: fig. 1B), the most recent of which were inscribed by Sachs [1] inside white circles on some of the bones ([21]: fig. 1B). The additional “½” vertebra identified during this study (Fig. **1A** **_1_–D)** was stored separately in the same collection area, but bears no label to indicate its anatomical position, taxonomic referral, or source locality. Despite this, the mottled color patterning and preservational condition (especially the characteristic diagenetic compression [1], [17]) is identical to the type material of *E. platyurus* (compare with Fig. 2A–D). There are likewise compatible tool marks left by mechanical preparation in the 19th century. Furthermore, the “½” centrum is morphologically and ontogenetically indistinguishable from the cranial-most cervicals of ANSP 10081, which represents an osteologically mature elasmosaurid [1]. **(1) Dimensions**. The “½” centrum fragment measures 49 mm long, by 45 mm high, and 47 mm wide across the articular facet. When complete, its proportions would therefore have been longer than either high or wide, as are the craniad cervicals of *E. platyurus* [1] (Fig. 2A, B). The presence of elongate cervical centra is usually considered diagnostic for Elasmosauridae [17], [19], [23], [24], [25], [26], [27], [28], [29], [30], although, the trait is known to vary taxonomically [31] with the articular faces being broader than centrum length in *Aphrosaurus* and *Thalassomedon* [17], *Libonectes morgani* [18], *Callawayasaurus* [32], and *Aristonectes* [33]. **(2) Articular surfaces**. The preserved articular surface on the “½” centrum (Fig. 1D) is concave, suggesting amphicoely, and bears both a pronounced, thickened rim and ventral notch. Flattened ( = platycoelous or acoelous [28]) cervical centra constitute an unambiguous synapomorphy for Elasmosauridae [27], [28], [29], [30], yet amphicoelous craniad cervicals have been documented in *E. platyurus* [1] (Fig. 2A) and *Albertonectes* [20] amongst North American latest Cretaceous taxa. The presence of a ventral notch is also considered indicative of advanced elasmosaurids (*sensu* [34]), but occurs elsewhere in basal plesiosauroids [25]), and differs from some Early Cretaceous forms including *Callawayasaurus* [32] and *Eromangasaurus* [35], [36], which tend to lack this feature. **(3) Lateral centrum surfaces**. The “½” centrum has markedly concave lateral sides (Fig. 1A_1_, A_2_), similar to ANSP 10081 (Fig. 2B, C), which is probably a result of crushing [1], [17]. Nevertheless, a pronounced lateral longitudinal ridge is still evident (Fig. 1A_1_), and represents another classic synapomorphy for Elasmosauridae (e.g. [17], [23]). The lateral ridges of elasmosaurids (or “keels” *sensu* [19], [23]) are usually pronounced in the craniad cervical series, but become reduced towards the caudal part of the neck in some taxa (e.g. *Mauisaurus*, *Hydrotherosaurus, Thalassomedon*, and *Libonectes* [17], [18], [37]); conversely, *E. platyurus* [1], *Styxosaurus snowii* [19], [28], and *Albertonectes* [20] retain prominent lateral ridges even in the caudad cervical region. Recent phylogenies have also advocated multiple independent acquisitions of lateral longitudinal ridges on the cervical centra within Plesiosauria [26], [28], [29], [34], thus correlating the trait with increased neck length in other plesiosauroids (e.g. *Occitanosaurus*, *Muraenosaurus*, *Spitrasaurus* [25], [26], [38]). **(4) Ontogeny**. Brown [23] established that co-ossification of the cervical centra with their corresponding ribs and neural arches, as well as a rounded articular surface rims, were reliable indicators of osteological maturity in plesiosaurians. Sachs [1] identified similar conditions in ANSP 10081, and broken remnants of fused rib/neural arch facets (Fig. 1A_1_, C), and thickened and rounded articular surface edges (Fig. 1D) are also detectable on the “½” centrum, which we conclude is probably part of the same individual.

## The Cervical-Dorsal Transition in Plesiosaurians

The initial identification of discrete vertebral morphotypes within the plesiosaurian axial skeleton is often attributed to Harry Govier Seeley [39], who in 1874 formally established the presence of a transitional sequence, termed “pectorals”, graduating the position of the lateral rib facet from the centrum body, upwards across the neurocentral suture, and onto the transverse process of the neural arch. This demonstrable structural and nomenclatural subdivision has since been adopted in nearly all publications on plesiosaurian osteology. Yet despite its ubiquitous usage, Carpenter ([12]: p. 150) proposed abandonment of the term because vertebrae manifesting a “rib facet [that] bridges the centrum-neural arch suture” and “single-headed ribs” had not been similarly differentiated in the pectoral region of extant lepidosaurians (Carpenter [12] cited Hoffstetter and Gasc [40] for a supporting example of this condition in *Sphenodon*). Carpenter [12] additionally stated: “it [thus] seems pointless and disadvantageous to [distinguish pectorals] in plesiosaurs” and “undue weight [has been placed] on this character phyletically”. Certainly, descriptive recognition of the plesiosaurian pectoral series has not been universal historically, with some researchers applying it inconsistently [41], only informally [42], or ignoring it altogether [43]. The pectoral vertebrae have also typically not been differentiated in basal sauropterygians (see [44] for synopsis), although, as noted by Lin and Rieppel ([45]: p. 9) the transition between the cervical and dorsal sequences is unclear in reptiles generally (like Carpenter [12] these authors cited Hoffstetter and Gasc [40] for evidence). Nevertheless, “the transition from double-headed cervical ribs to single-headed dorsal ribs can be used as a mark to differentiate the two regions” – at least in those cases where an obvious disjunctive transformation takes place (see [46] for a negative example). Where this is not apparent, the dorsal region has been designated as “start[ing] from the vertebra where the pectoral girdle is attached” [47]. However, this definition is problematic for plesiosaurians because the pectoral girdle is positioned ventrally and has no traceable connection with the vertebral column [48]. Romer [49] reported that the “posterior end of the [cervical] series” in nothosaurians (basal eusauropterygians *sensu* [44]) possessed “transitional segments” in which the diapophysis (“upper articular surface” *sensu* [49]) sequentially transferred to the neural arch while the parapophysis either fused with the diapophysis or disappeared. Carpenter ([12]: p. 150) nonetheless pointedly mentioned that neither Carroll [50] nor Storrs [51] used the term “pectorals” to define this intermediate morphology. Paradoxically, though, Carroll [50] never actually discussed subdivisions within the nothosaurian vertebral column, and Storrs ([51]: p. 22) did employ “pectoral” (with a comment on its occasionally ambiguous application) to describe the “transitional” position of the transverse process on “vertebra 19″ of *Corvosaurus*. Sues [52] also specifically referred to the pectorals in pistosauroids, the closest relatives of plesiosaurians [53], [54], nominating them as those vertebrae in which the functional rib facets are borne by both the neural arch and centrum. This alternately distinguished the pectorals from the last cervical where the parapophyses were still separated, and the first dorsal, characterized by complete removal of the rib facet to the transverse process of the neural arch ([52]: p. 119). Dalla Vecchia [55] accordingly recognized the pectoral vertebrae of pistosauroids (explicitly rejecting the opinions of Carpenter [12]) via their single rib facet transecting the neurocentral suture. He additionally noted that the transverse process of the succeeding dorsals incorporated a connection with the centrum (quote “the transverse process is formed largely or completely by the neural arch” [55]: p. 212). Note that by positional implication this should still accommodate the entire rib facet. In contrast, Sander *et al*. [56] were unable to pinpoint discrete pectorals in *Augustasaurus* (the immediate sister taxon of Plesiosauria [53], [54]), and thus correlated these vertebrae with the dorsals based upon their single rib articulation. Sato *et al*. ([53]: p. 183) conversely ascribed the pectorals in the most completely known basal pistosauroid, *Yunguisaurus*, to the cervical column despite this component of the skeleton being “obscured” in the holotype, and only visible in ventral view on one other published specimen ([54]: p. 4); the cervical sequence in this latter fossil was identified as “[t]he ventral edge of the rib facet [being] located on the centrum until the 50^th^ vertebra, but unclear in the 51^st^ [pectoral], and [then the] entire facet [restricted to the] neural arch from the 52^nd^ and after”.

Elimination of the “pectorals” as a discrete vertebral morphotype has caused substantial confusion in plesiosaurian phylogenetics. Most significantly, it has introduced ambiguity into the state designations for cervical and dorsal vertebral number, as well as overall neck length. For example, O’Keefe ([26]: p. 49, character 111) explained that his scores for cervical count excluded the pectorals, whose rib articulations did not arise exclusively from the centrum body. Druckenmiller and Russell ([28]: p. 52, character 99), on the other hand, avoided inclusion of the pectorals in their cervical vertebra number but instead described them as dorsals, and employed a quantitative coding that was sensitive to minor changes in unit value because it assigned separate states to each numerical subdivision (i.e. an increase of even one vertebra could substantially alter the scores). This impacted on their qualitative coding of relative neck length ([28]: p. 53, character 100) – as being “longer (0), or shorter (1), than the trunk”, which apparently involved the pectorals but excluded the sacrals from the dorsal series; this is despite the “trunk” (“*truncus*” *sensu Nomina Anatomica Veterinaria* 2012) incorporating the entire axial column (sacral region included) minus the neck (pectoral region exempted) and tail. Ketchum and Benson ([29]: Appendix 3, p. 21, character 118) contrastingly reinstated the pectorals into their cervical vertebra counts, and considered neck length to be partially dependent upon this character ([29]: Appendix 3, character X66). They also found that a meristic increase in cervical vertebrae number was reconstructed at their tree node uniting Elasmosauridae, Cryptocleididae, and Plesiosauridae ([29]: p. 385). Most recently, Benson and Druckenmiller [34] created an arbitrary division of both the cervical ([34]: Appendix 2, character 152) and dorsal column ([34]: Appendix 2, character 179) into sub-sets of two−15 vertebrae each, and used “dorsalised” rib morphology, together with “the location of vertebrae relative to the pectoral girdle” to identify the cervical series. Nevertheless, how this accommodated for taphonomic displacement of the pectoral girdle was not specified, and if disarticulated, the pectoral vertebrae were supposedly integrated into the dorsal series irrespective of their original life position. Moreover, the pectorals were then also analyzed separately via their own qualitatively scored (and interdependent) character ([34]: Appendix 2, character 180), which drew on an earlier conclusion [57] that the pectoral sequence could be identified by the rib facet comprising “portions of both the centrum and neural arch” (*sensu* [39]), and that this “can [variably] form part of the caudal cervical or cranial dorsal series” in different taxa.

**Figure 3:**
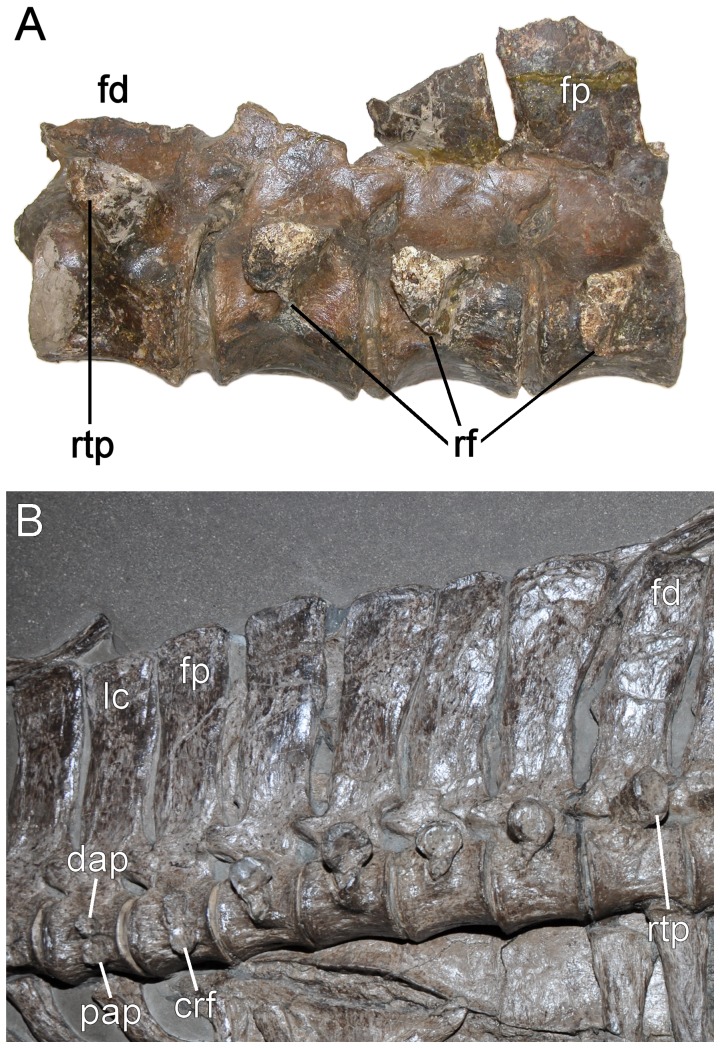
Pectoral series. A. *Elasmosaurus platyurus* (ANSP 10081) and B. *Seeleyosaurus guilelmiimperatoris* (Museum für Naturkunde Berlin, MB.R.1992). Not to scale. **Abbreviations.** **crf** = conjoint rib facet, **dap** = diapophysis, **fd** = first dorsal, **fp** = first pectoral, **lc** = last cervical, **pap** = parapophysis, **rf** = single rib facet, **rtp** = single rib facet on transverse process.

To counter these seemingly random redefinitions, together with what we feel is the off-handed disregard of a long-standing, morphologically accurate expression to describe a phylogenetically meaningful trait, we propose the reinstatement of “pectoral” into standard terminological usage as the most correct and convenient solution. Carpenter’s [12] original critique that the term is “pointless and disadvantageous” because it has not been applied to lepidosaurians is superfluous, since Plesiosauria is both independently divergent and unanimously monophyletic [26], [28], [29], [34], [53], [54], thus manifesting its own suite of uniquely derived features with recognizable intermediate conditions in ancestral lineages (e.g. nothosaurians and pistosauroids [49], [51], [52], [55]). The acquisition and evolutionary modification of a discrete pectoral series within the presacral vertebral column is therefore demonstrably evident (see Fig. 3), and essentially not contested, whereas the problem of practical definition relative to the cervical-dorsal transition is. Carpenter’s ([12]: p. 150) literal designations of the last cervical as “the vertebra in which the rib facet (formed by the combined diapophysis and parapophysis) extends across the centrum-neural-arch boundary” and is located “near the base of the of the neural canal”, and the first dorsal as “the vertebra in which the rib facet overlies the neural arch-centrum suture”, are both inadequate and counterintuitive because multiple consecutive vertebrae within the pectoral series could fit these definitions (e.g. the cervical terminus could be interpreted as part of the cranial dorsal region, or the first dorsal mistakenly construed as a cervical based on the rib facet “overlying” the neurocentral suture). The recommended use of the neural canal base as a proxy landmark for the fully fused vertebral sutures in osteologically mature individuals [12] is also problematic, since the neurocentral contact can extend well below the pedicle and even overly the cervical ribs as thin “lappets” in some taxa (e.g. *Hauffiosaurus* [29], [58]). Additionally, we question the use of pectoral girdle positioning as a determinant of vertebral placement [34] because there is no way of unambiguously establishing whether the appendicular elements are preserved in life position. Finally, Kubo *et al*. [20] advocated the presence of “long ribs” (presumably equating to the “dorsalised” category of Benson and Druckenmiller [34]) to distinguish the first dorsal, but this is subjective for disarticulated remains in which comparative rib proportions must be assumed (if they can be reconstructed at all: e.g. ANSP 10081 does not preserve complete ribs [1], [21]).

In contrast to these proposals, we therefore recommend a return to the primary data source – the pectoral vertebrae themselves – as designators of the transitional segment between the cervical and dorsal components of the vertebral column in plesiosaurians. This avoids any ambiguity imposed by inference from disarticulated and/or displaced limb girdle and rib components (*sensu* [20], [34]), and also eliminates the potential for character dependence (e.g. [34]) or descriptive conflict within phylogenetic assessments (e.g. [26], [28], [29], [34]). To clarify the pectorals morphologically, we define them as usually three or more distinctive vertebrae within the cranial forelimb girdle region that interpolate between the cervical and dorsal series. They bear a functional rib facet transected by the neurocentral suture, and conjointly formed by both the parapophysis on the centrum body and diapophysis from the neural arch (irrespective of rib length) (Fig. 3). This morphology is unambiguously distinguishable from the standard cervicals, in which the functional rib facet is borne exclusively on the centrum (irrespective of whether the neural arch projects a small ventral “lappet” that slightly overlaps the cervical rib: see [29], [58]), and the dorsals in which the rib articulation is situated above the neurocentral suture and functionally borne only by the transverse process of the neural arch (see Fig. 3B).

## Conclusion: Implications for the “Longest-necked” Plesiosaurians

Taking into account Cope’s missing “½” vertebra, as well as our revised definition for the plesiosaurian pectoral series, we emend the presacral vertebral count provided by Sachs [1] for the ANSP 10081 individual to 72 cervicals (including the atlas-axis complex), three pectorals, and potentially, at least 16 dorsals incorporating the additional vertebrae from FHSM VP-398 and KUVP 129744 identified by Everhart [21] (although see Noè and Gómez-Pérez [59] for counter-conjecture on the attribution of these supplementary specimens). The cervical-dorsal transition can be observed to occur progressively through the pectoral series (see Fig. 3A), with a single dorsoventrally elongate rib facet situated at the approximate level of the neurocentral suture (fused due to osteological maturity [1]) in vertebra 73, that sequentially compresses and tapers ventrally in vertebrae 74–75 following reduction of the parapophysis, while the diapophysis concomitantly expands and extends laterally onto the transverse process in vertebra 76– the first dorsal. The identified sequence of 72 cervicals in ANSP 10081 is also probably not complete, since the cranial section of the column was disarticulated and dispersed by erosion, and some vertebrae are known to have gone missing after the initial excavation [21], [59]. In spite of this, *Elasmosaurus platyurus* still manifests one of the highest numbers of cervical vertebrae recorded for any vertebrate, a phenomenon shared only with one other elasmosaurid, *Albertonectes vanderveldei* from the Upper Campanian Bearpaw Formation of Alberta, Canada [20]. The cervical series of *A. vanderveldei* reportedly comprised 76 vertebrae (including the atlas-axis complex) with the last signified by “the neural arch [forming] the dorsal rim of the rib facet and [beginning] to extend laterally from the side of the vertebra” ([20] p. 561). Because the rim of the rib facet contributes to its functional contact surface, we suggest that this vertebra is actually part of the pectoral series. Likewise, the succeeding 77^th^ and 78^th^ vertebrae display rib facets that extended across both the centra and neural arches, and thus morphologically comply with pectorals; note that Kubo *et al*. ([20] p. 561) designated these vertebrae as the dorsals because of their “long ribs”. Given our revised interpretation, we recognize 75 cervicals in the articulated column of *A. vanderveldei*, which is only three vertebrae longer than the incomplete cervical count of *E. platyurus*. Irrespectively, both of these plesiosaurians represent, to our current knowledge, the “longest-necked” animals ever to have lived, and belong to a clade (Elasmosauridae) that developed one of the most extreme structural specializations yet evidenced in vertebrate history.

## References

[1] Sachs S (2005) Redescription of *Elasmosaurus platyurus* Cope 1868 (Plesiosauria: Elasmosauridae) from the Upper Cretaceous (lower Campanian) of Kansas, U.S.A. Paludicola. 5: 92–106.

[2] Cope ED (1868) Remarks on a new enaliosaurian, *Elasmosaurus platyurus*. Proc. Acad. Nat. Sci. Philadelphia 20: 92–93.

[3] Cope ED (1869) Synopsis of the extinct Batrachia and Reptilia of North America, Part I. Transactions of the American Philosophical Society. 14: 1–235.

[4] Leidy J (1870) [Remarks on *Elasmosaurus platyurus*]. Proc. Acad. Nat. Sci. Philadelphia 22: 9–10.

[5] Leidy J (1870) On the *Elasmosaurus platyurus* of Cope. Am J Sci 49: 392.

[6] Spamer EE, Daeschler E, Vostreys-Shapiro LG (1995) A study of fossil vertebrate types in The Academy of Natural Sciences of Philadelphia; Taxonomic, systematic, and historical perspectives. Academy of Natural Sciences of Philadelphia, Special Publication 16: 1–434.

[7] Everhart MJ (2005) Oceans of Kansas - A Natural History of the Western Interior Sea. Indiana University Press, 322 pp.

[8] Davidson JP (2002) Bonehead mistakes: The background in scientific literature and illustrations for Edward Drinker Cope’s first restoration of *Elasmosaurus platyurus*. Proc. Acad. Nat. Sci. Philadelphia 152: 215–240.

[9] Marsh OC (1870) [Remarks on the remains an extinct bird allied to the turkey from the Greensand of Monmouth, N. J.]. Proc. Acad. Nat. Sci. Philadelphia 22: 11.

[10] Ballou WH (1890) [Statements by O. C. Marsh regarding the ‘head on the wrong end *Elasmosaurus*]. New York Herald 19: 11 (January 19, 1890)..

[11] Cope ED (1870) On *Elasmosaurus platyurus* Cope. Am J Sci 50: 140–141.

[12] Carpenter K (1999) Revision of North American elasmosaurs from the Cretaceous of the Western Interior. Paludicola 2: 148–173.

[13] Storrs GW (1999) An examination of Plesiosauria (Diapsida: Sauropterygia) from the Niobrara Chalk (Upper Cretaceous) of central North America. University of Kansas Paleontological Contributions 11: 1–15.

[14] Everhart MJ (2006) The occurrence of elasmosaurids (Reptilia: Plesiosauria) in the Niobrara Chalk of Western Kansas. Paludicola 5: 170–183.

[15] Cope ED (1870) (Cover date August, 1869). Synopsis of the extinct Batrachia and Reptilia of North America. Transactions American Philosophical Society 14: 1–252.

[16] Williston SW (1906) North American plesiosaurs: *Elasmosaurus*, *Cimoliasaurus*, and *Polycotylus* . Am J Sci 4: 221–236.

[17] Welles SP (1943) Elasmosaurid plesiosaurs with a description of the new material from California and Colorado. University of California Memoirs 13: 125–254.

[18] Welles SP (1949) A new elasmosaur from the Eagle Ford Shale of Texas. Fondren Science Series 1: 1–28.

[19] Welles SP (1952) A review of the North American Cretaceous elasmosaurs. University of California Publications in Geological Sciences 29: 44–143.

[20] Kubo T, Mitchell MT, Henderson DM (2012) *Albertonectes vanderveldei*, a new elasmosaur (Reptilia, Sauropterygia) from the Upper Cretaceous of Alberta. Journal of Vertebrate Paleontology 32: 557–572.

[21] Everhart MJ (2005) Elasmosaurid remains from the Pierre Shale (Upper Cretaceous) of western Kansas. Possible missing elements of the type specimen of *Elasmosaurus platyurus* Cope 1868? PalArch 4: 19–32.

[22] Druckenmiller PS, Russell AP (2006) A new elasmosaurid (Reptilia: Sauropterygia) from the Lower Cretaceous Clearwater Formation, northeastern Alberta, Canada. Paludicola 5: 184–199.

[23] Brown DS (1981) The English Upper Jurassic Plesiosauroidea (Reptilia) and a review of the phylogeny and classification of the Plesiosauria. Bulletin of the British Museum (Natural History), Geology Series 35: 253–347.

[24] Brown DS (1993) A taxonomic reappraisal of the families Elasmosauridae and Cryptoclididae (Reptilia, Plesiosauroidea). Revue de Paléobiolgie 7: 9–16.

[25] Bardet NP, Godefroit P, Sciau J (1999) A new elasmosaurid plesiosaur from the Lower Jurassic of Southern France. Palaeontology 42: 927–952.

[26] O’Keefe FR (2001) A cladistic analysis and taxonomic revision of the Plesiosauria (Reptilia: Sauropterygia). Acta Zoologica Fennica 213: 1–63.

[27] Sato T (2002) Description of plesiosaurs (Reptilia: Sauropterygia) from the Bearpaw Formation (Campanian–Maastrichtian) and a phylogenetic analysis of the Elasmosauridae. Ph.D. dissertation, Department of Geology and Geophysics, University of Calgary, Calgary, 391 pp.

[28] Druckenmiller PS, Russell AP (2008) A phylogeny of Plesiosauria (Sauropterygia) and its bearing on the systematic status of *Leptocleidus* Andrews, 1922. Zootaxa 1863: 1–120.

[29] Ketchum HF, Benson RBJ (2010) Global interrelationships of Plesiosauria (Reptilia, Sauropterygia) and the pivotal role of taxon sampling in determining the outcome of phylogenetic analyses. Biol Rev Camb Philos Soc 85: 361–392. 20002391 10.1111/j.1469-185X.2009.00107.x

[30] Vincent P, Bardet N, Suberbiola XP, Bouya B, Amaghzazm M, et al (2011) *Zarafasaura oceanis* a new elasmosaurid (Reptilia: Sauropterygia) from the Maastrichtian phosphates of Morocco and the palaeobiogeography of latest Cretaceous plesiosaurs. Gondwana Research 19: 1062–1073.

[31] O'Keefe FR, Hiller N (2006) Morphologic and ontogenetic patterns in elasmosaur neck length, with comments on the taxonomic utility of neck length variables. Paludicola 5: 206–229.

[32] Welles SP (1962) A new species of elasmosaur from the Aptian of Colombia and a review of the Cretaceous plesiosaurs. University of California Publications in Geological Sciences 44: 1–96.

[33] O'Gorman JP, Gasparini Z, Salgado L (2013) Postcranial morphology of *Aristonectes* (Plesiosauria, Elasmosauridae) from the Upper Cretaceous of Patagonia and Antarctica. Antarct Sci 25: 71–82.

[34] Benson RBJ, Druckenmiller PS (2013) Faunal turnover of marine tetrapods during the Jurassic-Cretaceous transition. Biol Rev Camb Philos Soc DOI: 10.1111/brv.12038. 10.1111/brv.12038 23581455

[35] Kear BP (2005) A new elasmosaurid plesiosaur from the Lower Cretaceous of Queensland, Australia. Journal of Vertebrate Paleontology 25: 792–805.

[36] Sachs S (2005) *Tuarangisaurus australis* sp. nov. (Plesiosauria: Elasmosauridae) from the Lower Cretaceous of northeastern Queensland, with additional notes on the phylogeny of the Elasmosauridae. Mem. Queensl. Mus. 50: 425–440.

[37] Hiller N, Mannering AA, Jones GM, Cruickshank ARI (2005) The nature of *Mauisaurus haasti* Hector, 1874 (Reptilia: Plesiosauria). Journal of Vertebrate Paleontology 25: 588–601.

[38] Knutsen EM, Druckenmiller PS, Hurum JH (2012) Two new species of long-necked plesiosaurians (Reptilia: Sauropterygia) from the Upper Jurassic (Middle Volgian) Agardhfjellet Formation of central Spitsbergen. Norwegian Journal of Geology 92: 187–212.

[39] Seeley HG (1874) On *Muraenosaurus leedsi*, a plesiosaurian from the Oxford Clay. Part 1. J Geol Soc London 30: 197–208.

[40] Hoffstetter R, Gasc J-P (1969) Vertebrae and ribs of modern reptiles. In: Gans C, editor. Biology of the Reptilia. Volume 1. Morphology A. London and New York: Academic Press. 201–310.

[41] Swinton WE (1947) Plesiosaurs in the City Museum, Bristol. Proceedings of the Bristol Naturalists’ Society 27: 343–360.

[42] Storrs GW (1997) Morphological and taxonomic clarification of the genus *Plesiosaurus*. In: Callaway JM, Nicholls EL, editors. Ancient Marine Reptiles. San Diego: Academic Press. 145–190.

[43] Tarlo LB (1960) A review of the Upper Jurassic pliosaurs. Bulletin of the British Museum (Natural History), Geology Series 4: 147–189.

[44] Rieppel O (2000) Sauropterygia I. Placodontia, Pachypleurosauria, Nothosauroidea, Pistosauroidea. In: Wellnhofer P, editor. Handbuch der Paläoherpetologie. Part 12A. München: Verlag Dr. Friedrich Pfeil. 1–134.

[45] Lin K, Rieppel O (1998) Functional morphology and ontogeny of *Keichousaurus hui* (Reptilia, Sauropterygia). Fieldiana (Geology) 39: 1–35.

[46] Cheng Y-N, Wu X-C, Sato T, Shan H-Y (2012) A new eosauropterygian (Diapsida, Sauropterygia) from the Triassic of China. Journal of Vertebrate Paleontology 32: 1335–1349.

[47] Liu J, Rieppel O, Jiang D-Y, Aitchison JC, Motani R, et al (2011) A new pachypleurosaur (Reptilia: Sauropterygia) from the lower Middle Triassic of southwestern China and the phylogenetic relationships of Chinese pachypleurosaurs. Journal of Vertebrate Paleontology 31: 292–302.

[48] Nicholls EL, Russell AP (1991) The plesiosaur pectoral girdle: the case for a sternum. Neues Jahrbuch für Geologie und Paläontologie Abhandlungen 182: 161–185.

[49] Romer AS (1956). Osteology of the Reptiles. Chicago: The University of Chicago Press, 772 pp.

[50] Carroll RL (1981) Plesiosaur ancestors from the Upper Permian of Madagascar. Philos Trans R Soc London. Series B 293: 315–383.

[51] Storrs GW (1991) Anatomy and relationships of *Corosaurus alcovensis* (Diapsida: Sauropterygia) and the Triassic Alcova Limestone of Wyoming. Bulletin of the Peabody Museum of Natural History 44: 1–163.

[52] Sues H-D (1987) Postcranial skeleton of *Pistosaurus* and interrelationships of the Sauropterygia (Diapsida). Zool J Linn Soc 90: 109–131.

[53] Sato T, Cheng Y-N, Wu X-C, Li C (2010) Osteology of *Yunguisaurus* Cheng et al., 2006 (Reptilia; Sauropterygia), a Triassic pistosauroid from China. Paleontological Research 14: 179–195.

[54] Sato T, Zhao L-J, Wu X-C, Li C (2013) A new specimen of the Triassic pistosauroid *Yunguisaurus*, with implications for the origin of Plesiosauria (Reptilia; Sauropterygia). Palaeontology DOI: 10.1111/pala.12048.

[55] Dalla Vecchia FM (2006) A new sauropterygian reptile with plesiosaurian affinity from the Late Triassic of Italy. Rivista Italiana di Paleontologia e Stratigrafia 112: 207–225.

[56] Sander PM, Rieppel OC, Bucher H (1997) A new pistosaurid (Reptilia: Sauropterygia) from the Middle Triassic of Nevada and its implications for the origins of plesiosaurs. Journal of Vertebrate Paleontology 17: 526–533.

[57] Benson RBJ, Evans M, Druckenmiller PS (2012) High diversity, low disparity and small body size in plesiosaurs (Reptilia, Sauropterygia) from the Triassic-Jurassic boundary. PLoS ONE 7(3): e31838. 22438869 10.1371/journal.pone.0031838 PMC3306369

[58] Benson RBJ, Ketchum HF, Noè LF, Gómez-Pèrez M (2011) New information on *Hauffiosaurus* (Reptilia, Plesiosauria) based on a new species from the Alum Shale Member (Lower Toarcian: Lower Jurassic) of Yorkshire, UK. Palaeontology 54: 547–571.

[59] Noè LF, Gómez-Pèrez M (2007) Postscript to Everhart, M.J. 2005. “Elasmosaurid remains from the Pierre Shale (Upper Cretaceous) of western Kansas. Possible missing elements of the type specimen of *Elasmosaurus platyurus* Cope 1868?” – PalArch’s Journal of Vertebrate Palaeontology 4, 3: 19–32. PalArch 2: 1–9.

